# Hepatitis E virus seroprevalence among employees in catering and public place industries in Nanjing, China

**DOI:** 10.1017/S0950268823001097

**Published:** 2023-07-13

**Authors:** Jinlei Guo, Juansheng Zhang, Lingling Li, Xingli Gao

**Affiliations:** 1 Microbiological laboratory, Qinhuai District Centre for Disease Control and Prevention, Nanjing, China; 2 Microbiological laboratory, Xi’an Centre for Disease Control and Prevention, Xi’an, China

**Keywords:** seroprevalence, hepatitis E virus, immunoglobulin M, alanine aminotransferase, RNA

## Abstract

This study aimed to assess the prevalence of anti-hepatitis E virus (HEV) immunoglobulin (Ig) M and elevated serum alanine aminotransferase (ALT) levels among employees in catering and public place industries. Blood samples were collected between January and December 2020 from 26,790 employees working in the Qinhuai district of Nanjing, China. Anti-HEV IgM in the serum samples was tested by the capture ELISA method and ALT was tested by the IFCC method. Samples positive for anti-HEV IgM or with ALT levels over 200 U/L were subjected to PCR screening of HEV RNA. The overall seroprevalence of anti-HEV IgM was 0.41%, and the seroprevalence was slightly higher in males (0.47%) than in females (0.37%); however, the difference was not substantial (*p* = 0.177). Seroprevalence of anti-HEV IgM increased with age, reaching its peak level after 48 years of age. The prevalence of elevated ALT levels was 4.24%, and males exhibited a higher prevalence than females (6.78% vs 2.65%, *p* < 0.001). Prevalence of elevated ALT levels differed in age groups and the 26–36-year-old group had the highest rate of elevated ALT levels. Employees with elevated ALT levels had a higher prevalence of positive anti-HEV IgM than those with normal ALT (0.57% vs 0.31%, *p* < 0.001). Positive HEV RNA was detected in one anti-HEV IgM-negative employee with ALT higher than 200 U/L. In our study, all the HEV RNA-positive and IgM-positive individuals are asymptomatic, and a combination of ALT tests, serological methods, and molecular methods is recommended to screen asymptomatic HEV carriers and reduce the risk of transmission.

## Introduction

Hepatitis E is a common infectious disease with a global distribution that is spread mainly through the fecal-oral transmission of the hepatitis E virus (HEV). There are estimated to be 20 million HEV infections worldwide each year [1]. HEV is a non-enveloped, single-stranded, positive-sense RNA virus belonging to the Hepeviridae family. Currently, HEV are grouped into eight genotypes (HEV1–8), of which HEV1–4 are the most frequent genotypes causing infections in humans. HEV1 and HEV2 are restricted to humans and transmitted via the fecal-oral route, mainly occur in developing regions, and are associated with epidemic and sporadic hepatitis E infections. HEV3 and HEV4 infections mainly develop through zoonotic transmission, which are caused by close contact with infected animals (such as pigs, goats, and cats) or the consumption of contaminated food (most commonly raw or undercooked meat), and are common in industrialised countries [2]. In China, HEV1 was the most isolated genotype; however, HEV4 has overtaken HEV1 in terms of the frequency of isolation nationwide since the year 2000, probably due to improved sanitation and hygiene measures [3].

China used to be an endemic area for HEV; a massive outbreak of hepatitis E occurred in the Xinjiang Uygur Autonomous Region of China from 1986 to 1988, which was one of the world’s largest epidemics of hepatitis E with 119,280 cases and 707 deaths [4]. With improved sanitation and the introduction of vaccines, China is now a low HEV-endemic country, with an incidence of 1.36 reported cases/100000 inhabitants/year according to the National Notifiable Infectious Disease Report of 2020 [5]. However, most people infected with hepatitis E are asymptomatic and do not go to hospital for a physical examination, which means that the report may be underestimating the true infection rate of the virus. A recent investigation revealed a high incidence of sporadic HEV infections in Shanghai, China [6]. Hepatitis E is also a foodborne disease; food prepared by infected handlers may put consumers at risk for HEV infection. In 2018, HEV caused an outbreak among mechanical factory workers in China owing to the consumption of contaminated pig livers [7]. In addition to this, the person-to-person transmission of HEV was also confirmed during the outbreak [8]. Public places are where people gather to study, work, travel, rest, entertain, go shopping, and so on. Employees working in public places and in daily close contact with the public [9], such as people working in museums, shopping malls, hotels, cinemas, and barbershops, play an important role in the spread of the viruses. Since infections are mostly asymptomatic, asymptomatic HEV carriers can spread the viruses to susceptible populations during the acute infection period, leading to the emergence of HEV epidemics. Thus, the surveillance of HEV acute infections in those employees is important for HEV control.

Diagnosis of acute HEV infection usually depends on the detection of specific anti-HEV immunoglobulin (Ig) M antibodies in a person’s blood. However, the antibody test may leave out the suspected samples during the window period when the IgM is negative. Elevated serum alanine aminotransferase (ALT) levels, which reflect the evidence of liver injury, are helpful in the screening of the negative anti-HEV IgM samples [10]; hence, ALT tests are performed to improve the HEV detection rates in our study.

Currently, there is no routine HEV surveillance in hospitals in China, only monitoring of patients with liver disease. Recent investigations on HEV mainly focused on the IgG antibodies, which indicated previous infections [11], or focused on the seroprevalence of IgM among blood donors or pregnant women [12, 13]. Our study focused on the surveillance of acute HEV infections in catering and public place industry employees, trying to evaluate the risk of HEV epidemics and propose prophylactic interventions for the effective control of HEV.

## Methods

### Study subjects

Subject recruitment information for our programme was posted via internet, newspapers, and television advertising. During the period of January to December 2020, subjects who were ≥16 years and employed in catering and public place industries in Qinhuai district, the most densely populated district of Nanjing, eastern China, were included to detect recent infections of HEV. The screening programme relied on subjects volunteering to come forward of their own volition. The demographic information of all subjects, such as their age and sex, was collected according to their identity cards. For the subjects who tested positive for anti-HEV IgM or with ALT levels over 200 U/L, the face-to-face interviews were carried out by trained personnel to collect further information on symptoms (such as jaundice, nausea, fever, anorexia, etc), travel histories (whether they have been to HEV-epidemic areas in the past 15–75 days prior to the sample collection day), and specific job types. Informed consent was obtained from each subject. Ethical approval from the Ethical Committee of our institution had been obtained. The authors assert that all procedures contributing to this work comply with the ethical standards of the relevant national and institutional committees on human experimentation and with the Helsinki Declaration of 1975, as revised in 2008.

### Specimen collection, HEV IgM antibody, and ALT test

Five milliliters of venous blood were drawn from each subject. Blood samples were left for 3–5 hours at room temperature to allow clotting and then centrifuged to separate the serum for the test of HEV IgM antibody and ALT on the sample collection day. Commercially available ELISA kits (Wantai Biological Pharmacy Enterprise Co., Ltd, Beijing, China) were used to detect the anti-HEV IgM in serum samples. The positive criterion for anti-HEV IgM was based on the manufacturer’s instructions; optical density (OD) values were measured at 450 nm/630 nm. The cutoff value was determined based on the mean OD value of the negative control (NC) by the following formula: Cutoff value = 0.26 + NCmean. Test samples with OD values equal to or greater than the cutoff value indicated a positive sample. The serum biochemical parameter ALT was tested by the IFCC method using the ALT detection kits (Mindray Bio-Medical Electronics Co., Ltd, Shenzhen, China). According to the Chinese Health Industry Standard (WS/T404.1–2012), males over 50 U/L or females over 40 U/L were classified as individuals who featured elevated ALT levels. All the kits were within their validity period and used according to the manufacturer’s instructions.

### HEV RNA detection

Serum samples positive for anti-HEV IgM or with ALT levels >200 U/L between January and December 2020 were stored at −20 °C for the test of HEV RNA by real-time reverse transcription polymerase chain reaction (RT-PCR) at the beginning of October 2022. Here we adopt the ALT >200 U/L as the cutoff point for the HEV RNA test based on the previous studies [10, 14]. RNA was detected with a commercially available HEV RNA detection kit (ACON, Hangzhou, China) with the lowest detection limit of 500 copies/mL. Viral nucleic acids were extracted from 100 μL of serum. The RT-PCR included 20 μL viral nucleic acids, 0.6 μL primers and fluorescence probe mixture, 1.4 μL enzyme, and 18 μL RT-PCR mix. The RT-PCR amplification was carried on the LightCycler 480II real-time PCR instrument (Roche, Basel, Switzerland) based on the following condition: 50 °Cfor 20 min and 70 °Cfor 15 min, followed by 45 cycles of pre-denaturation step at 94 °C for 15 *s* and extension at 60 °Cfor 30 s. Fluorescence signal was collected at 60 °C. Samples that had evidence of amplification were subjected to a conventional RT-PCR, as previously described, to determine the genotype [6].

### Statistical analysis

IBM SPSS Statistics version 17.0 software (SPSS Inc., Chicago, IL, USA) was used for the statistical analysis. Categorical data were tested using Pearson’s chi-square (χ^2^) test and a two-sided *p-*value of less than 0.05 was considered statistically significant.

## Results

### Seroprevalence of anti-HEV IgM

A total of 26,790 catering and public place industry employees working in the Qinhuai district of Nanjing were enrolled in our study for the screening of anti-HEV IgM. They were aged between 16–79 years, with a median age of 36 years, and consisted of 16,432 (61.3%) females and 10,358 (38.7%) males.

**Figure 1.**
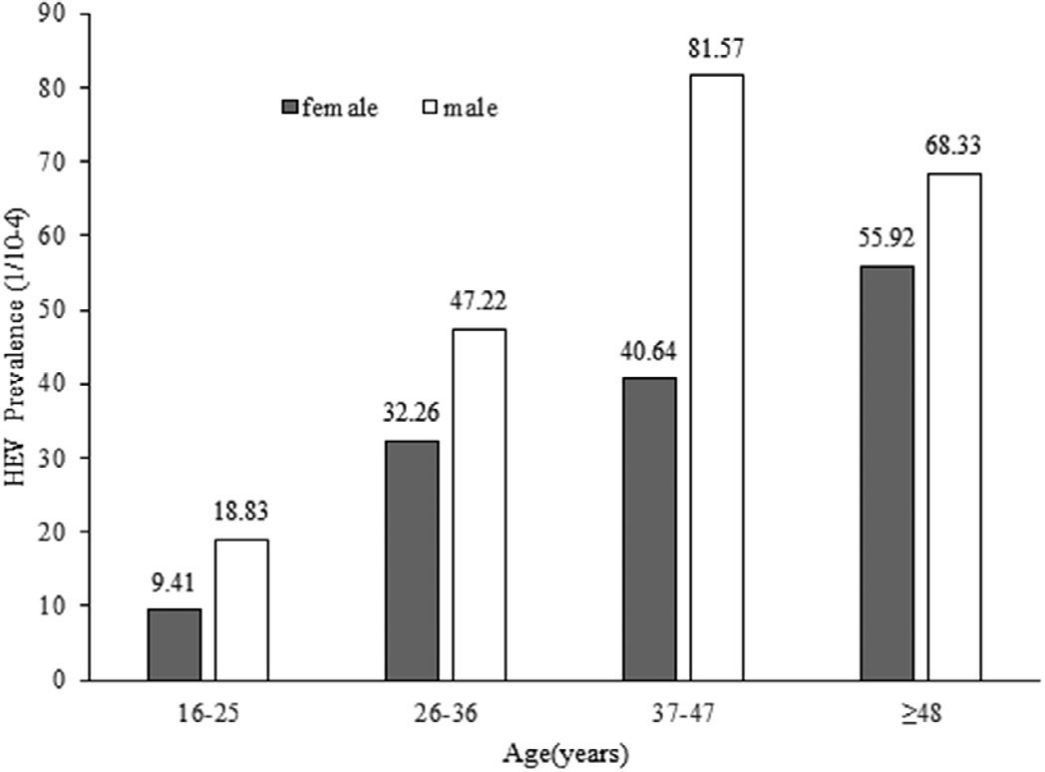
Age and gender distribution of hepatitis E prevalence among males (open block) and females (shaded block).

In total, 109 employees were tested positive for anti-HEV IgM antibodies, which corresponds to a seroprevalence of 0.41% (95% confidence interval [CI], 0.34–0.49). The seroprevalence revealed a statistically significant increase with study-subject age by means of the Chi-square test for linear trend (χ^2^ = 18.599, *p* = 0.000) (Table 1). The seroprevalence of anti-HEV IgM was slightly higher in males (0.47%, 49/10358) than in females (0.37%, 60/16432); however, the difference was not substantial (χ^2^ = 1.826,*p* = 0.177) (Table 1) (Figure 1).

**Table 1.** Prevalence of hepatitis E virus and elevated ALT levels by gender and age among 26790 employees in Nanjing, 2020

	Sample size	Elevated ALT level				Anti-HEV IgM positive		
		N	Prevalence (%)	*p*-value		N	Prevalence (%)	*p*-value
Gender								
Male	10358	702	6.78	<0.001		49	0.47	0.177
Female	16432	435	2.65			60	0.37	
Age (years)								
16–25	6906	282	4.08	0.004		10	0.14	0.000
26–36	6783	340	5.01			26	0.38	
37–47	6760	270	3.99			35	0.52	
≥48	6341	245	3.86			38	0.60	
Total	26790	1137	4.24			109	0.41	

*Note*: The percentages (%) were calculated by dividing the number of subjects with elevated ALT level or seropositive for IgM by the total number of subjects tested in each gender or age group.

Abbreviations: ALT = alanine aminotransferase; Anti-HEV IgM = Hepatitis E virus immunoglobulin M antibody; Elevated ALT level = males higher than 50 U/L or females higher than 40 U/L.

All the 109 subjects with positive HEV IgM were asymptomatic and 11.0% (12/109) of them had elevated ALT levels. The age of the positive subjects ranged from 17 to 67 years, with a median age of 42 years, consisting of 49 males and 60 females. 48.6% (53/109) of them were employed in the catering industry. 35.8% (39/109) of the positive subjects were females of childbearing age (18–49 years old). None of the females with positive HEV IgM were pregnant at the time of the blood collection. In our study, a total of 12,591 subjects were females of childbearing age (18–49 years old), and among them, 39 females were HEV IgM positive; While the number of the rest of study population was 14,199, of which 70 subjects were HEV IgM positive. Statistical analysis showed that HEV positivity in females in the childbearing age (39/12591, 0.31%) is lower than that in the rest of the study population (70/14199, 0.49%) (χ^2^ = 5.53, *p* = 0.019).

### The correlation of elevated serum ALT levels and the hepatitis E infection

The ALT is abundant in liver cells, and elevated serum ALT levels that reflect the evidence of liver injury can be used to improve the HEV detection rates. Our result showed 4.24% (1137/26790) of the employees were identified with elevated ALT levels (males higher than 50 U/L or females higher than 40 U/L) according to the reference intervals for common clinical biochemistry tests in China (WS/T 404.1–2012). Elevated ALT levels varied from 41 U/L to 424 U/L. The prevalence of elevated ALT levels was significantly higher in males than in females (6.78% vs 2.65%, χ^2^=266.662, *p* < 0.001). Statistics analysis showed that the prevalence of elevated ALT levels differed in different age groups, of which the 26–36-year-old group had the highest rate of elevated ALT levels (Table 1). Positive HEV IgM was detected both in employees with elevated ALT levels and in those with normal ALT. In the elevated ALT group, 12 samples were positive for anti-HEV IgM, corresponding to a prevalence of 1.05% (12/1137). While among the 25,653 serum samples with normal ALT, 97 (0.38%) samples were positive for anti-HEV IgM. Employees with elevated ALT levels had a higher prevalence of anti-HEV IgM than those with normal ALT (1.05% vs 0.38%, χ^2^=12.325, *p* < 0.001).

### PCR detection of HEV RNA

The serum samples positive for anti-HEV IgM or with ALT levels over 200 U/L were subjected to real-time RT-PCR screening of HEV RNA.

Of the 109 anti-HEV IgM-positive subjects, 105 serum samples tested for HEV RNA were RNA negative, while HEV RNA tests of the remaining 4 subjects were not performed because of limited serum samples.

There are 14 samples with elevated ALT levels higher than 200 U/L in our study, of which 11 samples were subjected to HEV RNA screening (the remaining 3 samples did not have enough serum to do the test), and among the tested, one sample was RNA positive. The RNA-positive employee was anti-HEV IgM negative and asymptomatic with an elevated ALT level of 312 U/L, indicating that she was in the window period of HEV acute infection. With no history of recent travel in HEV-endemic areas or eating raw meat, she worked in a fast food restaurant and prepared raw meat as part of her daily work; besides, she kept a pet cat in her house. Unfortunately, due to the low viral load in the serum sample, we were unable to obtain the sequences and determine the genotype.

## Discussion

Located in the Yangtze River Delta region of China, Nanjing is the provincial capital of Jiangsu, of which Qinhuai district is the most densely populated with prosperous business and tourism, attracting large numbers of migrant workers and tourists each year. Here we investigate employees in the catering industry and those working in public places in the Qinhuai district, who interact with the public daily, and try to provide a view of the recent infections of HEV in the employees and help evaluate the risk of the HEV epidemics in the local residents.

Anti-HEV IgM is the first antibody that appears after HEV infection; the positive IgM is an important marker of recent HEV infection and is increasingly used in routine diagnosis of hepatitis E since its reliability has been dramatically increased by using antigens with immunodominant epitopes of HEV [15]. The overall prevalence of HEV IgM for the investigated employees in Nanjing was 0.41%. In order to gain a better analysis of the prevalence of anti-HEV IgM in our area, we reviewed recently published representative reports from domestic and international regions, as listed in Table 2. The prevalence of anti-HEV IgM in different studies has a high heterogeneity, ranging from 0.41% to 3.8%. This discrepancy in the prevalence of anti-HEV IgM may be influenced by different test systems with varying sensitivities, effects of subjects selection such as exposure to contaminated food sources (raw meat), or contact with certain animal species (pigs, wild boars, and so on), study areas with varying levels of hygiene, and local sanitation infrastructure. Additionally, the discrepancy in seroprevalence results across locations could be attributed to the actual intensity of HEV infection and circulation in each geographic area. Our investigation indicated that investigated employees in Nanjing had a low seroprevalence of anti-HEV IgM, which is comparable to study populations in Italy and Portugal [16, 17], and is lower than the swine workers of Guangzhou [18] or healthy individuals of Nigeria [19], or the general populations (1.8%) in mainland China, according to a meta-analysis [20]. Nanjing is a developed city with high socioeconomic status and good sanitary conditions, which may explain the low HEV infection rate of the investigated employees. Although our results showed the HEV seroprevalence was higher in men than in women, it was not considered statistically significant, similar to some previous studies [17, 19, 21], whereas other reports provided a contrasting result [13]. Thus, whether HEV infection exhibits gender bias or not needs further verification. None of the positive anti-HEV IgM samples tested in our study had detectable RNA, and similar observations have been described previously in some studies [13, 22, 23]. We speculate there are several possibilities: firstly, the subjects in our study are apparently healthy individuals without symptoms and the blood samples might be taken in the convalescent period when the viremia subsides, as the period of viremia lasts very short, whereas IgM, which is produced early in the acute phase, can persist for a relatively longer time until and after the infection resolves [24]. Secondly, HEV RNA was detected retrospectively in our study and RNA in stored serum may degrade over time. Besides, some subjects might be reactive to anti-HEV IgM non-specifically due to unknown reasons. Although the RNA is the direct evidence of acute infection, there is evidence that shows HEV of 20%–30% of the infected persons are cleared when symptoms develop [25]; thus the negative RNA results cannot exclude the acute infection. Our results show the likelihood of contracting HEV increases with age, reaching a peak level after 48 years of age, which are in accord with the previous investigations [19, 26]. The aging of the population is becoming more and more serious globally, the elderly tend to have a variety of chronic diseases such as diabetes, hypertension, and heart diseases, and have a higher chance of developing severe hepatitis E. It is worth noting that 35.8% of the anti-HEV IgM-positive study subjects are females of childbearing age in our study; infections in pregnant women usually have more severe consequences [2]. This underscores the importance of vaccination and surveillance of HEV in the elderly and childbearing-age women who are at high risk.

**Table 2.** Studies on seroprevalence of HEV IgM in China and worldwide in recent reports

Study	Region	Year^a^	Subjects	Number	Prevalence
Our data	Nanjing, China	2020	Catering or public place industry employees who are≥16 years	26790	0.41%
[12]	InnerMongolia, China	2018	Pregnant women who visited the Inner Mongolia Maternal and Child Care hospital in 2018	3278	0.6%
[18]	Guangzhou, China	2015–2016	Swine workers who are ≥16 years and exposed to pigs over 5 h/week for over 10 weeks are included and those who are immunocompromised or pregnant or acute respiratory tract infected are excluded	332	2.71%
[13]	Dali, China	2018	Blood donors who visited the Dali Blood Center from February to March 2018	1864	1.13%
[23]	Southern Brazil	2013–2018	risk populations (crack users, low-income area residents, cirrhotic and liver transplant patients) who did not donate blood recently and healthy blood donors who are≥18 years	400	1.5%
[21]	Shiraz, Iran	2011–2012	healthy populations without ongoing infectious diseases	1030	0.9%
[16]	Italy	2017-2019	blood donors	7172	0.5%
[17]	Portugal	2015-2016	general populations	1656	0.48%
[19]	Osun State, Nigeria	2015–2017	apparently healthy individuals from 6 communities in Osun State	653	3.8%

a The year of sampling.

The ALT and HEV RNA testing are combined to screen HEV in the window period of negative IgM in our study. We adopt the ALT > 200 U/L as the cutoff point for the HEV RNA test based on the previous studies. Positive HEV RNA was detected in one anti-HEV IgM negative employee with elevated ALT of 312 U/L among the tested 11 employees with ALT levels higher than 200 U/L; however, we cannot determine the genotype due to the low viral load of the serum sample. The routes of HEV transmission of the RNA-positive employee are not clear as she had no history of recent travel to HEV-endemic areas or eating raw meat, and we speculate that the occupational contact with raw pork meat might result in her infection, as the HEV is a zoonotic virus.

Consistent with the results of previous studies [27], our results reveal that the male gender or a younger age group (26–36-year-olds) is associated with a high likelihood of elevated serum ALT levels. It has been shown that high estradiol levels and estrogen receptors in the liver can protect females from acute liver injury [28]. Besides, lifestyle factors causing liver injury, such as drinking alcohol and staying up late, are common among modern young people in urban China, with men more likely to engage in heavy drinking [29, 30]. Taken together, this may partly help explain why males and the younger age group were found to have a higher prevalence of elevated ALT levels. However, other associated risk factors that cause liver injury deserve further investigation. The two groups should receive screening for liver function more regularly in order to avoid serious liver injury.

There were some limitations in our study. First, the epidemiological data of the study subjects were scarce. Therefore, we could not comprehensively evaluate the risk factors. Second, the job types of the majority of the subjects were not distinguished between catering industries and public place industries when they were enrolled, and the lack of this information disabled us from separating out the seroprevalence by type of industry and assessing the effects of job types on the HEV infection, which we will improve in the future investigations. Besides, we could not determine the HEV genotypes circulating in the local area as we could not obtain the virus sequences. However, it is possible that the most prevalent strains circulating among the local residents are genotype 4, according to a previous report on Nanjing [31].

In China, the hepatitis E vaccine was licensed in 2012. In 2020, only about 100,000 HEV vaccines were issued by National Institutes for Food and Drug Control in China (https://bio.nifdc.org.cn/pqf/search.do), suggesting a very low HEV vaccination rate among Chinese people. A recent meta-analysis shows that the overall prevalence of HEV IgG seropositivity was only 27.3% among the general population in mainland China [20]. The anti-HEV IgM seroprevalence (0.41%) of the subjects indicates a low HEV endemicity state in Nanjing, China. However, possible epidemics may break out once there are imported HEV cases since most of the local individuals are antibody-naïve due to the lack of naturally acquired immunity and the vaccination of HEV. In our study, all the HEV RNA-positive and IgM-positive individuals are asymptomatic and may act as a major source of transmission; thus a comprehensive surveillance of HEV that combines ALT tests, serological methods, and molecular methods is recommended to screen asymptomatic virus carriers and help propose prophylactic interventions for effective control of HEV.

## Data Availability

The data that support the findings of this study are available upon request from the corresponding author.
